# Does postoperative cognitive decline after coronary bypass affect quality of life?

**DOI:** 10.1136/openhrt-2020-001569

**Published:** 2021-04-22

**Authors:** Fredrike Blokzijl, Frederik Keus, Saskia Houterman, Willem Dieperink, Iwan C C van der Horst, Michiel F Reneman, Anthony R Absalom, Massimo A Mariani

**Affiliations:** 1 Department of Cardiothoracic Surgery, University Medical Centre Groningen, Groningen, The Netherlands; 2 Department of Critical Care, University Medical Centre Groningen, Groningen, The Netherlands; 3 Department of Education and Research, Catharina Hospital, Eindhoven, The Netherlands; 4 Department of Intensive Care, Maastricht University Medical Centre, Maastricht, The Netherlands; 5 Department of Rehabilitation Medicine, University Medical Centre Groningen, Groningen, The Netherlands; 6 Department of Anesthesiology, University Medical Centre Groningen, Groningen, The Netherlands

**Keywords:** coronary artery bypass, atherosclerosis, outcome assessment, healthcare

## Abstract

**Objective:**

This study aimed to explore the influence of coronary artery bypass grafting (CABG) on both postoperative cognitive dysfunction and quality of life (QoL) and the association between the two patient-related outcomes.

**Methods:**

In a prospective, observational cohort study, patients with elective, isolated CABG were included. Cognitive function was assessed using the Cogstate computerised cognitive test battery preoperatively, 3 days and 6 months after surgery. QoL was measured preoperatively and at 6 months using the RAND-36 questionnaire including the Physical Component Score (PCS) and the Mental Component Score (MCS). Regression analysis, with adjustment for confounders, was used to evaluate the association between postoperative cognitive dysfunction and QoL.

**Results:**

A total of 142 patients were included in the study. Evidence of persistent cognitive dysfunction was observed in 33% of patients after 6 months. At 6 months, the PCS had improved in 59% and decreased in 21% of patients, and the MCS increased in 49% and decreased in 29%. Postoperative cognitive changes were not associated with QoL scores.

**Conclusions:**

Postoperative cognitive dysfunction and decreased QoL are common 6 months after surgery, although cognitive function and QoL were found to have improved in many patients at 6 months of follow-up. Impaired cognitive function is not associated with impaired QoL at 6 months.

**Trial registration number:**

NCT03774342.

**Key questions**

**What is already known about this subject?**

- Postoperative cognitive dysfunction (POCD) is a common neurocognitive disorder with an incidence of 30%–60% after coronary artery bypass grafting. Despite the high incidence of POCD, the effect of POCD on patients’ daily lives after coronary bypass has not yet been investigated.

**What does this study add?**

- This study showed that most patients benefit in terms of an increased quality of life (QoL) and recovery of cognitive functioning 6 months after surgery. However, long-term POCD was still present in one out of three patients and QoL was not yet at the preoperative level in a large proportion of patients 6 months after surgery. Impaired cognitive function was not associated with impaired QoL.

**How might this impact on clinical practice?**

- These findings enable healthcare workers to tailor the care and guidance of patients during the process of shared decision making prior to surgery. Patients’ preferences and expectations on postoperative recovery need to be discussed thoroughly during the preoperative phase. Studies on patient-related outcomes can be valuable during this counselling process.

## Introduction

In the past few decades, improvements in operative techniques and perioperative care have led to a steady decline in mortality after cardiac surgery. Although survival rates have improved, elderly patients are at increased risk of postoperative complications such as neurological and pulmonary problems.^1 2^ Neurological complications after cardiac surgery have been classified by the American College of Cardiology and the American Heart Association into two categories.^3^ Type I deficits result from well-defined local or regional insults resulting in transient ischaemic attack (TIA), stroke, coma and fatal brain injury. Type II deficits result from more diffuse and poorly understood insults, and include delirium and postoperative cognitive dysfunction (POCD). Delirium is clearly defined in the *Diagnostic and Statistical Manual for Mental Disorders*, Fifth Edition.^4^ In contrast, the definition and operationalisation of POCD is less clear; it is mostly described as a deficit of concentration, attention, memory and motor speed that lasts for several weeks or months.^3^ Recently, an expert working group produced a set of recommendations for diagnosis and nomenclature for postoperative neurocognitive disorders to align the terminology used with that of the DSM-V.^5^ They recommended that the term POCD be used for mild or major neurocognitive disorders found to be present between 1 and 12 months after surgery. Studies of patients who have undergone coronary artery bypass grafting (CABG) describe an incidence of POCD of 30%–60% depending on the timing, type and interpretation of cognitive tests used, and the patient population involved.^6 7^ Despite this high incidence and the fact that in vulnerable elderly patients even a small decline may have important consequences such as loss of independence,^8 9^ data on the impact of POCD on quality of life (QoL) are scarce. The primary aim of this study was to explore the influence of CABG on cognitive function and QoL, and the secondary aim was to investigate the association between POCD and QoL in adult patients after CABG.

## Methods

We conducted a prospective single-centre cohort study. The study protocol is registered at ClinicalTrials.gov. This article describes the cognitive outcomes of our study population in relation to QoL; outcomes on physical performance, as described in the study protocol, will be reported in another article. The study results are reported according to the Strengthening the Reporting of Observational Studies in Epidemiology guidelines^10^ (online supplemental material S1).

10.1136/openhrt-2020-001569.supp1 Supplementary data

### Eligibility criteria

We included adult patients admitted for elective, solitary on-pump CABG in the University Medical Centre Groningen, the Netherlands. Exclusion criteria were previous cardiac surgery and combined surgery (due to the increased risks of complications), pre-existing neurological deficits (ie, dementia, stroke and epilepsy) and psychiatric illness limiting reliability of screening tests. If patients were likely to experience difficulty completing cognitive testing due to impaired eyesight or hearing, or problems understanding the Dutch language, they were also excluded from the study.

### Procedures

On the day of admission, usually the day before surgery, patients were identified and contacted for informed consent by the attending doctor or nurse practitioner. After informed consent was obtained, patients were included and baseline preoperative measurements were obtained. Postoperative assessment of cognitive function was performed in the hospital 3 days after surgery (short term) and at the patients’ homes 6 months after surgery (long term). QoL was measured at baseline and 6 months after surgery.

### Demographic and medical characteristics

Baseline demographic data were retrieved from the electronic patient medical records and included age, sex, Body Mass Index, education level (low: primary school; moderate: high school or secondary vocational education; high: college (applied sciences) or university), EuroSCORE I and II, and the presence of comorbidity such as diabetes,^11^ pulmonary disease,^11^ arterial vascular disease,^11^ renal disease^12^ and impaired ventricular function.^13^ Definitions of comorbidities are included in online supplemental material S2. Perioperative data included duration of surgery, time of cardiopulmonary bypass, cross-clamp time (in minutes) and the number of (arterial) grafts.

10.1136/openhrt-2020-001569.supp2 Supplementary data

### Outcome measures

Cognitive function was assessed using the Cogstate brief computerised cognitive test battery (Cogstate, Melbourne, Australia). The test battery we used consisted of four tasks: the detection task, the identification task, the one card learning task and the one back task (ONB), assessing psychomotor speed, selective attention, visual learning and working memory, respectively.^14^ The Cogstate tests have been used in several other studies, indicating a good sensitivity for detecting subtle changes in cognitive performance and strong test–retest reliability.^14 15^ On the day before surgery, the tests were performed twice as recommended by the software vendor. The first was to minimise practice effects and the second was used as baseline test. Before starting, each task was introduced by the researcher using standardised written instruction. Each set of four tests required approximately 20 min to complete. All cognitive function scores were standardised according to normative data from age-matched controls.^16^ A standardised score higher than 100 indicated a better than average score compared with the age-matched population.^17^ As suggested by Evered *et al*,^5^ signs of cognitive dysfunction 3 days after surgery were interpreted as delayed neurocognitive recovery effected by drugs, anaesthesia and/or pain with the potential for recovery.

To perform a within-subject analysis a standardised reliable change Z-score for each postoperative cognitive test was calculated, based on the difference between the postoperative and baseline score, and normalised using test–retest variability data provided by the software vendor.^18^ The standardised change Z-scores of all four individual tasks were summed to generate a composite Z-score.^7^ POCD was operationally defined as a Z-score of <−2 in two or more individual tasks or a composite Z-score of <−2.^18^ This threshold of <−2 was chosen to provide consistency with the suggestion of the expert working party of defining POCD as equivalent to major neurocognitive disorder if the decline in test scores is >2 SDs.^5^

QoL was measured using the RAND-36 V.2 questionnaire. The questionnaire is a widely used and validated instrument containing eight health domains: physical functioning, social functioning, role limitations due to physical health problems, role limitations due to emotional problems, mental health, vitality, pain and general health perception.^19^ Each dimension is scored on a scale between 0 and 100; a higher score is equivalent to better health. Two summarised scores were calculated: a Physical Component Score (PCS) and a Mental Component Score (MCS). We considered a minimal clinically important difference to be 5 points and calculated the change in QoL for each patient between preoperative and postoperative measurements. QoL was judged as being improved (>5 points), worse (<5 points) or unchanged (≤5 points decrease or increase in score).^11^ Secondary outcomes were postoperative complications including delirium,^20^ atrial fibrillation,^21^ myocardial infarction,^22^ surgical re-exploration,^11^ deep sternal wound infection^11^ and renal failure,^11^ all within 30 days after surgery and stroke/TIA within 72 hours after surgery.^23^ Definitions of complications are included in online supplemental material S2. Additional outcomes were duration of stay at the intensive care unit (ICU) and discharge destination.

### Data analysis

The sample size calculation was based on the hypothetical association between POCD and QoL. POCD was assumed to be the independent variable and QoL as the dependent variable. A previous study on patients with POCD after CABG reported an SD of 8.5^17^ and a study on QoL after cardiac events reported an SD of 11.^24^ A sample size of 123 patients was required for a two-tailed test at a minimal detectable difference of 0.33, an α of 0.05 and power of 80% to detect an association between POCD and QoL. To account for missing data, we included 142 patients.

Characteristics of patients are presented as numbers (with percentages) for dichotomous variables and as means (with SD) or medians (with IQRs) for continuous variables depending on distributions. Differences between baseline and 6 months of follow-up of cognitive function and QoL were tested using paired t-tests. Differences in baseline, operative and postoperative characteristics between the patients with and without POCD were tested using the χ^2^ or Fisher’s exact test. Linear regression analysis was used to evaluate the impact of POCD on the difference in QoL (dependent variable). Possible risk factors for POCD based on literature, as well as age and baseline PCS/MCS, were included in the multivariable model. All analyses were tested two-sided, and tests with p values of <0.05 were considered statistically significant. All data were analysed using SPSS V.25.0.

## Results

A total of 142 patients undergoing elective CABG were enrolled between October 2018 and July 2019 (online supplemental material S3). Table 1 presents all baseline, operative and postoperative characteristics. Based on the standardised composite Z-score at baseline, two patients already had mild cognitive impairment based on a composite Z-score of >1 SD preoperatively.

10.1136/openhrt-2020-001569.supp3 Supplementary data

**Table 1:** Baseline, operative and postoperative characteristics of patients with CABG

	n=142
Baseline characteristics	
Sex (female)	18 (13)
Age (years), mean (SD)	64.3 (9.4)
BMI (kg/m^2^)	
<25	34 (24)
25–30	71 (50)
>30	37 (26)
Log EuroSCORE I, median (IQR) In groups	2.7 (1.9–4.7)
<10%	133 (94)
10%–20%	9 (6.3)
>20%	0 (0.0)
EuroSCORE II, median (IQR)	1.5 (1.1–2.2)
Diabetes mellitus	31 (22)
Pulmonary disease	15 (11)
Arterial vascular disease	7 (4.9)
Renal disease	14 (9.9)
LVEF	
>50%	96 (68)
30%–50%	45 (32)
<30%	1 (0.7)
Education level*	
Low	40 (28)
Moderate	55 (39)
High	46 (33)
Operative characteristics	
Number of grafts	
One graft	1 (0.7)
Two grafts	139 (98)
Three grafts	2 (1.4)
Number of arterial grafts	
Use of one arterial graft	100 (70)
Use of two or more arterial grafts	39 (27)
No arterial graft	3 (2.1)
Surgical time,† mean (SD)	254 (41)
CPB time,† mean (SD)	106 (30)
Cross-clamp time,† mean (SD)	64 (22)
Postoperative characteristics	
Delirium	7 (4.9)
Atrial fibrillation	14 (9.9)
Myocardial infarction	2 (1.4)
Surgical re-exploration	3 (2.1)
Deep sternal wound infection	3 (2.1)
Stroke/TIA	0 (0.0)
Renal failure	0 (0.0)
ICU stay,‡ median (IQR)	21 (18–25)
Discharge destination	
Home	99 (71)
Other hospital	21 (15)
Rehabilitation centre	18 (13)
Nursing home	1 (0.7)

Values are presented as n (%) unless otherwise indicated.

*Education level for one patient unknown.

†Surgical time, CPB time and cross-clamp time in minutes.

‡ICU stay in hours.

BMI, Body Mass Index; CABG, coronary artery bypass grafting; CPB, cardiopulmonary bypass; ICU, intensive care unit; LVEF, left ventricular ejection fraction; TIA, transient ischaemic attack.

**Figure 1:**
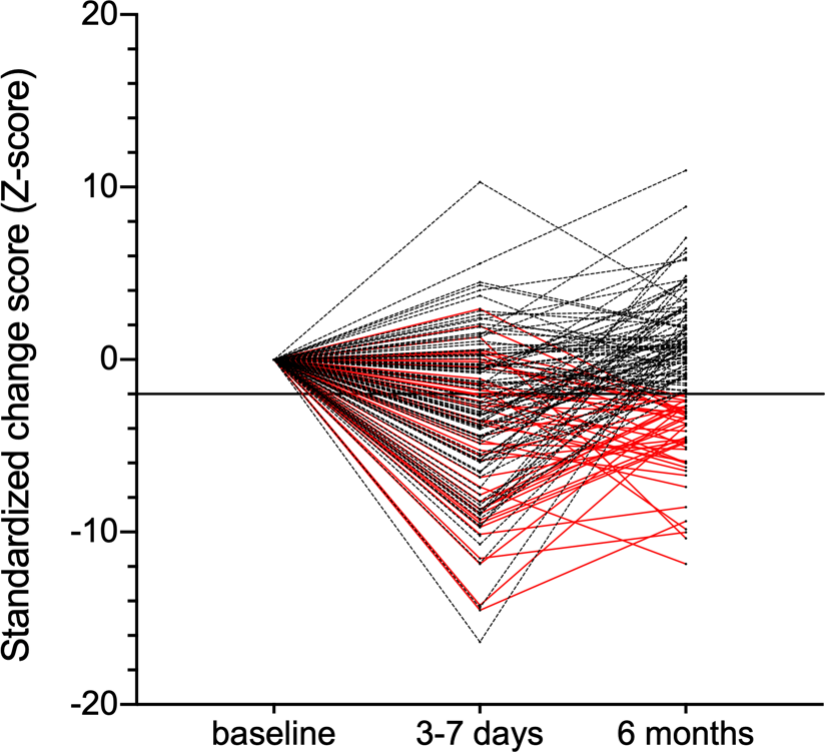
Development of the composite standardised cognitive change scores per patient over time. Red solid lines indicate patients who had Z-scores below −2 at 6 months. Black dashed lines indicate patients who had a Z-score above −2 at 6 months of follow-up.

Short-term postoperative cognitive tests were performed after a median of 3 days (range 3–7) after surgery. Three patients refused further participation, and five patients were unable to complete the early postoperative test due to pain or dizziness during testing (three patients), a prolonged stay in the ICU (one patient) and transfer to another hospital on day 3 (one patient). Among the remaining 134 patients, 80 patients (60%) fulfilled the criteria for early cognitive dysfunction 3 days after surgery, based on delayed neurocognitive recovery in the terminology suggested by Evered *et al*.^5^ Two patients died during follow-up; one patient moved abroad; and five patients refused further participation. Long-term cognitive tests were performed in 131 patients after a median of 192 days (range 177–219) after surgery. Forty-three patients (33%) had cognitive dysfunction at long-term follow-up. Twenty-nine patients (22%) showed improved cognitive function with a >2 increase in their cognitive function scores compared with baseline scores. Figure 1 shows the composite standardised cognitive change scores measured at 3–7 days and 6 months after surgery. The mean cognitive test scores are presented in online supplemental material S4.

10.1136/openhrt-2020-001569.supp4 Supplementary data

### Quality of life

**Figure 2:**
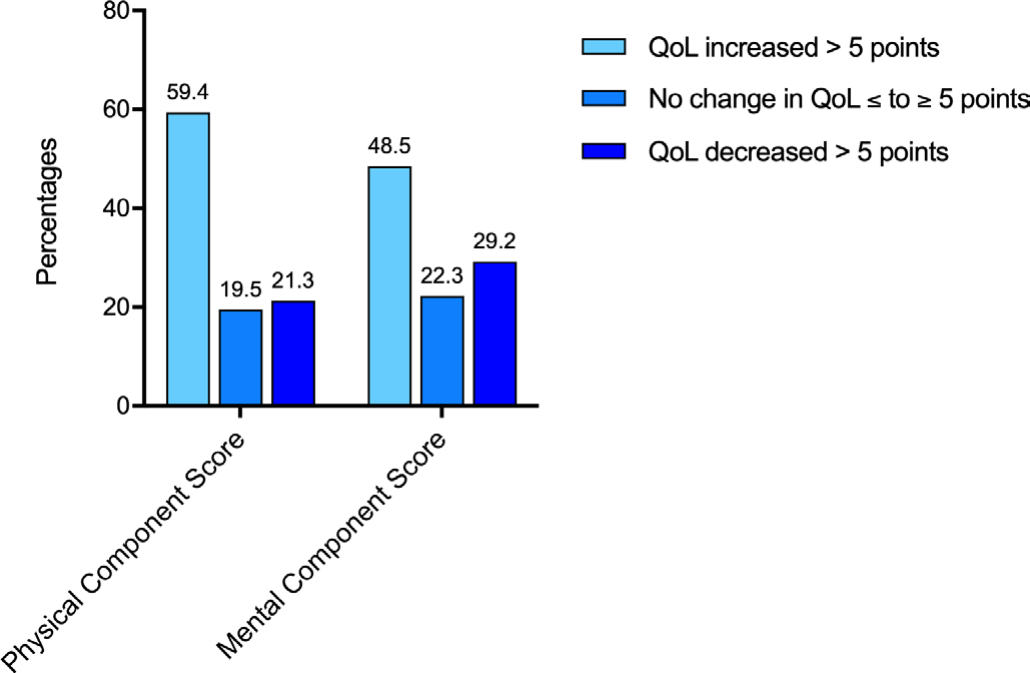
Differences between baseline and 6 months follow-up in the QoL of patients with coronary artery bypass grafting: Physical Component Score and Mental Component Score; cut-off value: 5 points. QoL, quality of life.

At 6 months of follow-up, PCS was increased (>5 points) in 59% of the patients and decreased (>5 points) in 21%. MCS was increased in 49% of the patients and decreased in 29% (figure 2). The mean MCS, PCS and subscale scores are presented in online supplemental material S5.

10.1136/openhrt-2020-001569.supp5 Supplementary data

### Association between postoperative cognitive dysfunction and QoL

Table 2 shows the results of the regression analysis. Associations between POCD and difference in QoL 6 months after surgery were non-significant (PCS p=0.66 and MCS p=0.91, respectively). The association between age and PCS did not reach statistical significance (p=0.06). In the multivariable analysis, baseline PCS and education level were statistically significantly correlated with difference in PCS at 6 months of follow-up. Baseline MCS was associated with the difference in MCS 6 months after CABG.

**Table 2:** Univariable and multivariable linear regression models of the effect of preoperative and postoperative factors on the difference in quality of life 6 months after coronary artery bypass grafting

Univariable analysis				Multivariable analysis		
PCS				PCS		R^2^=0.42
	Beta	95% CI	P value	Beta	95% CI	P value
POCD Z-score at 6 months	0.20	−0.68 to 1.07	0.66	0.18	−0.53 to 0.88	0.62
Age	−0.35	−0.71 to 0.02	0.06	−0.23	−0.52 to 0.07	0.13
Baseline PCS	−0.59	−0.73 to −0.45	<0.001	−0.59	−0.73 to −0.45	<0.001
Baseline cognitive functioning	7.83	−19.8 to 35.1	0.57	6.99	−15.3 to 29.3	0.54
Education level	4.50	0.17 to 8.84	0.04	5.00	1.52 to 8.48	0.05
Delirium	3.72	−12.3 to 19.8	0.65	−4.06	−17.5 to 9.45	0.55

MCS				MCS		R^2^=0.28
	Beta	95% CI	P value	Beta	95% CI	P value
POCD Z-score at 6 months	0.03	−0.71 to 0.76	0.94	−0.18	−0.85 to 0.50	0.61
Age	−0.10	−0.41 to 0.21	0.52	0.06	−0.23 to 0.34	0.69
Baseline MCS	−0.41	−0.53 to −0.29	<0.001	−0.43	−0.56 to −0.30	<0.001
Baseline cognitive functioning	−0.99	−32.1 to 14.1	0.44	−1.99	−23.4 to 19.4	0.86
Education level	0.16	−3.52 to 3.84	0.93	0.60	−2.69 to 3.89	0.72
Delirium	3.78	−9.78 to 17.3	0.58	−5.00	−18.0 to 7.97	0.45

MCS, Mental Component Score; PCS, Physical Component Score; POCD, postoperative cognitive dysfunction.

### Patients with and without POCD at 6 months of follow-up

Of 80 patients with POCD at short-term follow-up, 37 (46%) recovered their cognitive dysfunction 6 months after surgery. Forty-three patients (54%) were classified as having persistent POCD at long-term follow-up. Baseline characteristics, operative characteristics and postoperative complications of patients with and without POCD at 6 months after surgery are presented in table 3. Age (p=0.040), education level (p=0.046) and postoperative delirium (p=0.015) were different between the groups. Differences in PCS and MCS between the groups were not statistically significant (online supplemental material S6).

10.1136/openhrt-2020-001569.supp6 Supplementary data

**Table 3:** Characteristics of patients with or without POCD 6 months after CABG

	No POCD (n=88)	POCD (n=43)	P value
Baseline characteristics			
Sex (female)	10 (11)	5 (12)	>0.99
Age (years), mean (SD)	63 (9.2)	67 (9.0)	0.040
BMI (kg/m^2^)			0.29
<25	18 (20)	13 (30)	
25–30	49 (56)	18 (42)	
>30	21 (24)	12 (28)	
Log EuroSCORE I, median (IQR) In groups	2.6 (1.5–4.4)	3.6 (2.1–5.3)	0.72
<10%	83 (94)	40 (93)	0.15
10%–20%	5 (6.0)	3 (7.0)	
EuroSCORE II, median (IQR)	1.5 (1.1–2.2)	1.5 (1.1–2.5)	0.71
Diabetes mellitus	20 (2.3)	9 (20.9)	0.79
Pulmonary disease	7 (8.0)	7 (16.3)	0.23
Arterial vascular disease	4 (4.5)	3 (7.0)	0.68
Renal disease	8 (9.1)	5 (11.6)	0.76
LVEF			0.54
>50%	58 (66)	33 (36)	
30%–50%	29 (33)	10 (23)	
<30%	1 (1.1)	0 (0.0)	
Education level			0.046
Low	27 (31)	9 (21)	
Moderate	39 (44)	14 (33)	
High	22 (25)	20 (46)	
Baseline PCS, mean (SD)	65 (20)	63 (19)	0.46
Baseline MCS, mean (SD)	71 (20)	73 (21)	0.51
Cognitive test results, mean (SD)			
DET	101 (6.7)	103 (5.3)	0.24
IDN	100 (4.9)	101 (4.9)	0.69
OCL	104 (8.7)	104 (8.4)	0.71
ONB	98 (5.6)	98 (6.1)	0.49
Operative characteristics			
Surgical time,* mean (SD)	257 (46)	249 (33)	0.24
CPB time,* mean (SD)	108 (32)	101 (24)	0.21
Cross-clamp time,* mean (SD)	66 (23)	61 (22)	0.21
Postoperative characteristics			
Delirium	1 (1.1)	5 (12)	0.015
Atrial fibrillation	9 (10)	5 (12)	>0.99
Myocardial infarction	1 (1.1)	0 (0.0)	>0.99
Surgical re-exploration	2 (2.3)	0 (0.0)	>0.99
Deep sternal wound infection	2 (2.3)	0 (0.0)	>0.99
ICU stay†, median (IQR)	21 (18–25)	21 (17–23)	0.25
Discharge destination			0.99
Home	63 (72)	31 (72)	
Other hospital	12 (14)	6 (14)	
Rehabilitation centre	12 (14)	6 (14)	
Nursing home	0 (0.0)	0 (0.0)	

Values are presented as n (%) unless otherwise indicated.

*Surgical time, CPB time and cross-clamp time in minutes.

†ICU stay in hours.

BMI, Body Mass Index; CABG, coronary artery bypass grafting; CPB, cardiopulmonary bypass; DET, detection task; ICU, intensive care unit; IDN, identification task; LVEF, left ventricular ejection fraction; MCS, Mental Component Score; OCL, one card learning task; ONB, one back task; PCS, Physical Component Score; POCD, postoperative cognitive dysfunction.

## Discussion

In this prospective cohort study, we observed that many patients showed a postoperative improvement in cognitive function, and in the physical and mental component of QoL. However, POCD was persistent in 33% of patients 6 months after surgery, and there was either no change or a decline in QoL in approximately half of all patients. Contrary to our expectations, we did not find an association between POCD and difference in QoL at 6 months after CABG. A possible explanation could be that people with impaired cognitive function can still experience a high QoL. Alternatively, patients may adjust their perceived level of QoL (glad to be alive), so that the difference between the new and the intended level is normalised after surgery.

Many studies have been performed on POCD after CABG, mostly addressing the incidence and aetiology of POCD.^6 17 25^ The difficulty with studies on POCD are the lack of universally accepted definitions and gold standards for measuring POCD, sometimes leading to conflicting results.^6 9^ As in other POCD studies, we used the reliable change index that relates the change of scores to the normal test–retest variation in an age-matched control group.^7 17 18 25^ Other commonly used statistical methods are an absolute decline (usually >1 SD calculated from preoperative scores) or a percentage change from baseline (usually a decline of >20%). However, these methods do not relate to data from age-matched controls and therefore do not account for normal variability among a population.^26^ Recommendations about thresholds are published by the expert working group, but they do not recommend specific tests.^5^ The Cogstate test battery may be suitable as standard instrument because the major strength of this instrument is the comparability of retrieved data to data from age-matched controls. Also, several studies suggest this instrument to be both sensitive and reliable.^14–16^

The high number of patients in our study not improving after CABG indicates that CABG may have a high impact on patients’ QoL, as suggested by other studies.^27 28^ The long-term incidence of POCD in our study is also rather high, which is in line with other studies.^6 7^ Possible explanations for POCD include perioperative factors (ie, low blood pressure leading to altered cerebral perfusion, cerebral microemboli caused by disruption of aortic atherosclerotic plaques, anaesthesia and systemic inflammatory response) and patient-related factors (ie, systemic atherosclerosis and decline in cognitive performance caused by altered age). Current evidence suggests that on-pump and off-pump CABG procedures have similar long-term cognitive outcomes.^6 25 29^ One possible explanation is that some patients already have microinfarcts before surgery and another is that both CABG techniques cause new microinfarcts during surgery that are silent (ie, do not cause overall neurological deficits) but are sufficient to cause POCD. Studies to investigate this hypothesis could use diffusion-weighted MRI imaging before and after surgery to evaluate the correlation between microinfarct load and cognitive outcome.

Other explanations for the long-term decline in QoL and the high incidence of long-term POCD could be side effects of surgery (ie, new comorbidities or reduced independence) or other confounding factors unrelated to the intervention. Perhaps future studies should also assess functional status and work resumption alongside QoL to learn more about the impact of subtle changes in cognitive functioning on patients’ daily lives.

Risk factors for a decreased QoL after CABG identified in this study are high baseline PCS and MCS, suggesting that patients with a good QoL before surgery are more likely to experience a decreased QoL after surgery, also known as regression to the mean.^28 30^ Although not the primary outcome of our study, we found several significant differences between the groups with and without POCD. Age, education level and postoperative delirium have been identified as risk factors for POCD in other studies as well as in our study.^6 17^ Although our results indicate these variables as risk factors, our study groups are too small to reach for strong conclusions and should therefore be interpreted as hypothesis generating, or supportive of previous findings.

Our study has some important limitations. First, our patient selection might differ from other hospitals, which may limit generalisability, although we included only elective patients in our cohort and mortality risk was low, with a mean log EuroSCORE I of 3.8 (SD ±3). Second, it is likely that the early postoperative cognitive tests performed at 3 days after surgery were influenced by factors like sleep disturbance and opioids. We specifically chose day 3 for assessment of short-term POCD due to logistic reasons: many of our patients are transferred back to other hospitals on day 4 after surgery.

The main reasons to offer bypass surgery are to increase survival and symptom relief. Patient-reported outcomes like QoL and POCD are also important outcomes considered from the patients’ perspective. Our study shows high incidences of long-term POCD and a decreased QoL 6 months after CABG, which may negatively influence patients’ daily lives. Studies addressing these topics can provide valuable information for patients, relatives and doctors regarding shared decision making.

## Data Availability

All data relevant to the study are included in the article or uploaded as supplementary information. The data that support the findings in this study are available from the corresponding author (FB) on reasonable request.
