# Supplementary material for: Does postoperative cognitive decline after coronary bypass affect quality of life?
Source: Open Heart. 2021 Apr 22;8(1):e001569. doi: 10.1136/openhrt-2020-001569 (PMC8070880; doi:10.1136/openhrt-2020-001569)
Supplement: Supplementary data [file openhrt-2020-001569supp002.pdf]

Supplementary Material S2. Definitions of comorbidities & complications

Comorbidities

Diabetes: oral therapy or insulin dependent diabetes (11).

Pulmonary disease: prolonged use of steroids or other lung medication (11).

Arterial vascular disease: peripheral or abdominal vascular pathology or operation due to arterial vascular disease (11).

Renal disease: a reduced renal function prior to surgery with an estimated Glomerular Filtration rate (eGFR) <60 ml/min/1.73 m^2 (12).

Ventricular function: left ventricular ejection fraction good >50%, moderate 30-50% or poor <30% (13).

Complications

Delirium during hospital admittance defined as:

1) A Delirium Observation Screening (DOS)-score ≥ 3 at hospital ward and/or
2) A positive Confusion Assessment Method for the Intensive Care Method (CAM-ICU) score at the ICU and/or
3) Diagnosis confirmed by a psychiatrist or geriatrist according to the DSM-IV criteria (20).

Atrial fibrillation defined as new onset atrial fibrillation or atrial flutter requiring medical treatment or cardioversion within 30 days after surgery (21).

Myocardial infarction (MI) in the postoperative period. Myocardial infarction associated with CABG (within 48 hours after CABG) is arbitrarily defined by elevation of cardiac biomarker values >10 x 99th percentile upper reference limit (URL) in patients with normal baseline cardiac troponin values. In addition, either (i) new pathological Q waves or new LBBB, or (ii) angiographic documented new graft or

new native coronary artery occlusion, or (iii) imaging evidence of new loss of viable myocardium or new regional wall motion abnormality (22). After 48 hours, the standard definition of myocardial infarction is appropriate. The following criteria meets the diagnosis for MI: detection of a rise and/or fall of cardiac biomarker values, preferably cardiac troponin, with at least one value above the 99th percentile URL and in addition, either (i) symptoms of ischaemia, or (ii) new or presumed new significant ST-segment–T wave (ST–T) changes or new left bundle branch block (LBBB), or (iii) development of pathological Q waves in the ECG, or (iiii) imaging evidence of new loss of viable myocardium or new regional wall motion abnormality or identification of an intracoronary thrombus by angiography or autopsy (22).

Surgical re-exploration within 30 days after surgery: thoracotomy due to bleeding, cardiac tamponade or graft failure (11).

Deep wound infection within 30 days after surgery: when deeper tissues are affected (muscle, sternum and mediastinum) and one or more of the following three criteria are met:

1) surgical drainage or refixation

2) an organism is isolated from culture of mediastina tissue or fluid

3) antibiotic treatment because of a sternal wound (11).

Stroke: an acute neurological event within 72 hours after surgery with focal signs and symptoms and without evidence supporting any alternative explanation. Diagnoses of stroke requires confirmation by a neurologist (23).

Renal failure within 30 days after surgery when one or more of the following criteria are met:

1) renal replacement therapy (dialysis or CVVH) which was not present preoperatively
2) highest postoperative creatinine level > 177 µmol/L <u>and</u> a doubling of the preoperative value (the preoperative creatinine value is the value on which the EuroSCORE is calculated) (11).
