# Supplementary figures and images for: Does postoperative cognitive decline after coronary bypass affect quality of life?
Source: Open Heart. 2021 Apr 22;8(1):e001569. doi: 10.1136/openhrt-2020-001569 (PMC8070880; doi:10.1136/openhrt-2020-001569)

**Supplementary Material S3. CONSORT Flowchart Study population**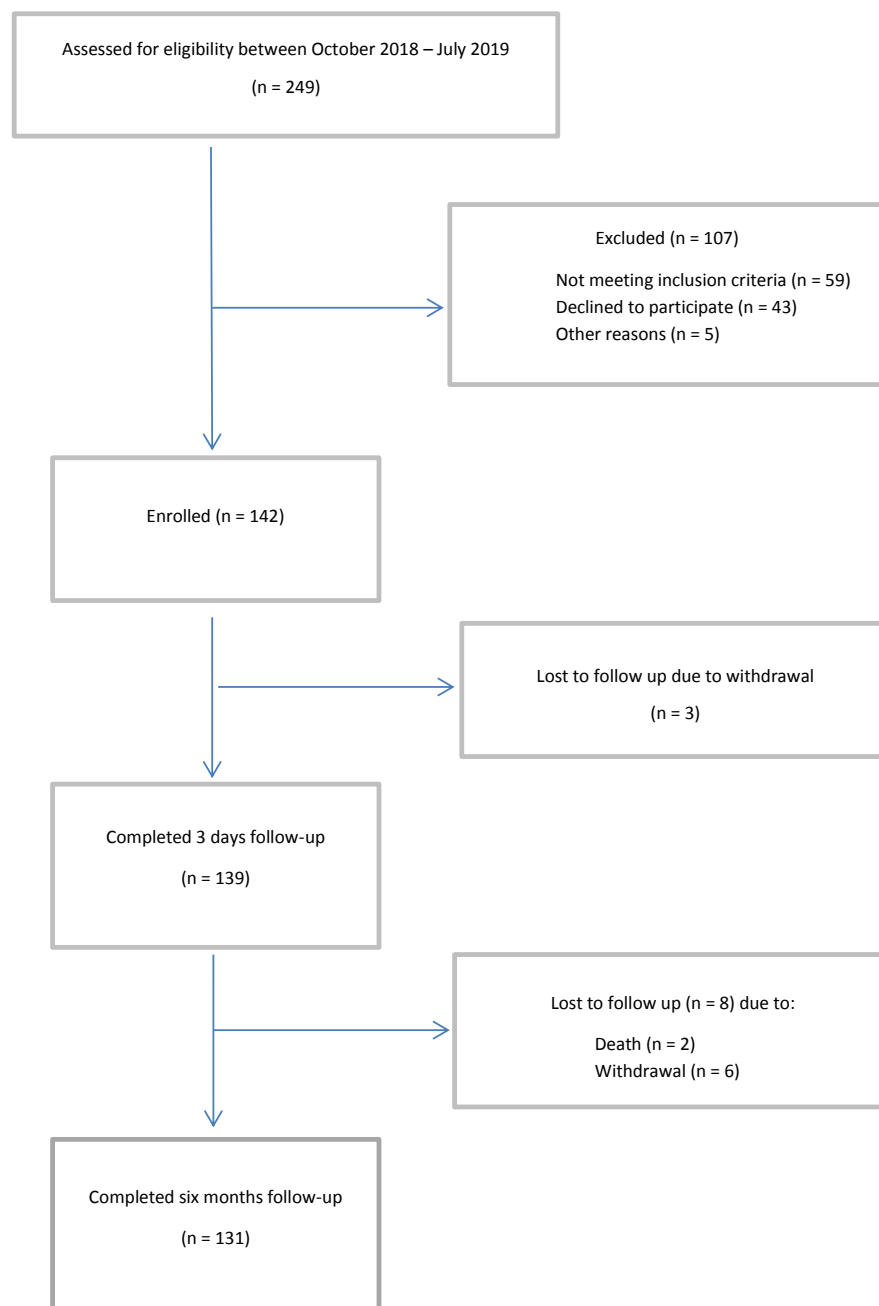

Supplement: Supplementary data [file openhrt-2020-001569supp003.pdf]
