# Supplementary material for: Does postoperative cognitive decline after coronary bypass affect quality of life?
Source: Open Heart. 2021 Apr 22;8(1):e001569. doi: 10.1136/openhrt-2020-001569 (PMC8070880; doi:10.1136/openhrt-2020-001569)
Supplement: Supplementary data [file openhrt-2020-001569supp004.pdf]

Supplementary Material S4. Standardised cognitive test-scores of patients before and after CABG

Test	Preoperative (n = 142)	3 days[1] (n = 134)	6 months (n = 131)	p-value[2]
DET; speed	101.8 ± 6.2	98.1 ± 8.4	100.5 ± 8.3	0.07
IDN; speed	100.6 ± 4.8	98.2 ± 6.3	100.8 ± 5.5	0.26
OCL; accuracy	103.6 ± 8.7	100 ± 9.0	104.5 ± 9.4	0.58
ONB; speed	98.3 ± 5.6	95.6 ± 6.2	97.5 ± 5.8	0.08

[1]Five patients did not complete the test. [2] paired T-test only from patients with complete dataset; P-value based on preoperative and 6 months scores. DET:detection task; IDN:identification task, OCL:one card learning task; ONB:one back task. All numbers are presented as mean with standard deviation.
