# Supplementary material for: Does postoperative cognitive decline after coronary bypass affect quality of life?
Source: Open Heart. 2021 Apr 22;8(1):e001569. doi: 10.1136/openhrt-2020-001569 (PMC8070880; doi:10.1136/openhrt-2020-001569)
Supplement: Supplementary data [file openhrt-2020-001569supp005.pdf]

**Supplementary Material S5.** Quality of life of patients before and after CABG –subscale scores

| Scores | Preoperative (n = 140) | 6 months (n = 131) | P value |
|---|---|---|---|
| **Physical component score** | **63.4 ±19.9** | **73.0 ±17.4** | **<0.001** |
| General health | 64.0 ±17.4 | 65.7 ±17.3 | 0.24 |
| Physical functioning | 64.9 ±27.1 | 79.4 ±20.2 | <0.001 |
| Role physical | 57.4 ±30.2 | 64.3 ±29.0 | 0.009 |
| Bodily pain | 70.5 ±25.1 | 83.1 ±20.5 | <0.001 |
| | | | |
| **Mental component score** | **70.6 ±20.4** | **74.6 ±18.7** | **0.018** |
| Mental health | 74.7 ±19.2 | 78.3 ±18.1 | 0.012 |
| Vitality | 61.6 ±23.9 | 63.6 ±19.9 | 0.29 |
| Social functioning | 74.3 ±26.7 | 81.6 ±21.2 | 0.001 |
| Role emotional | 74.6 ±25.7 | 75.5 ±26.9 | 0.68 |

All numbers are presented as mean with standard deviation.
For a few patients not all scores on all subscales are known.
