# Supplementary material for: Does postoperative cognitive decline after coronary bypass affect quality of life?
Source: Open Heart. 2021 Apr 22;8(1):e001569. doi: 10.1136/openhrt-2020-001569 (PMC8070880; doi:10.1136/openhrt-2020-001569)

Supplementary Material S6. Difference in QoL of CABG-patients with and without POCD: physical and mental component score

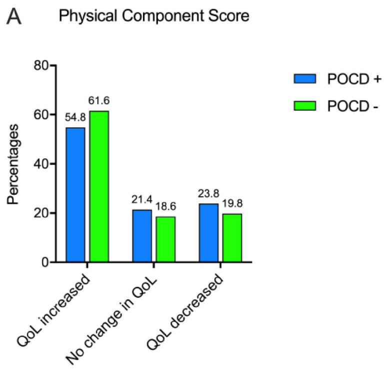

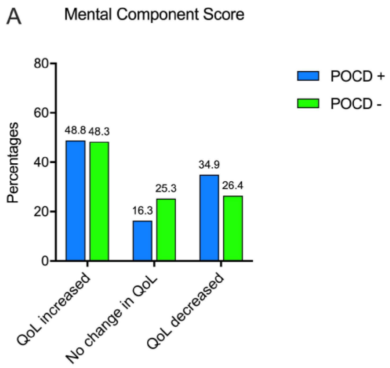

Supplement: Supplementary data [file openhrt-2020-001569supp006.pdf]
